# FSBC: fast string-based clustering for HT-SELEX data

**DOI:** 10.1186/s12859-020-03607-1

**Published:** 2020-06-24

**Authors:** Shintaro Kato, Takayoshi Ono, Hirotaka Minagawa, Katsunori Horii, Ikuo Shiratori, Iwao Waga, Koichi Ito, Takafumi Aoki

**Affiliations:** 1 NEC Solution Innovators, Ltd, 1-18-7 Shinkiba, Koto-ku, Tokyo, 136-8627 Japan; 2 Graduate School of Information Sciences, Tohoku University, 6-6-05 Aramaki Aza Aoba, Aoba-ku, Sendai-shi, Miyagi, 980-8579 Japan

**Keywords:** SELEX, Aptamer, Next-generation sequencing, Sequence analysis

## Abstract

**Background:**

The combination of systematic evolution of ligands by exponential enrichment (SELEX) and deep sequencing is termed high-throughput (HT)-SELEX, which enables searching aptamer candidates from a massive amount of oligonucleotide sequences. A clustering method is an important procedure to identify sequence groups including aptamer candidates for evaluation with experimental analysis. In general, aptamer includes a specific target binding region, which is necessary for binding to the target molecules. The length of the target binding region varies depending on the target molecules and/or binding styles. Currently available clustering methods for HT-SELEX only estimate clusters based on the similarity of full-length sequences or limited length of motifs as target binding regions. Hence, a clustering method considering the target binding region with different lengths is required. Moreover, to handle such huge data and to save sequencing cost, a clustering method with fast calculation from a single round of HT-SELEX data, not multiple rounds, is also preferred.

**Results:**

We developed fast string-based clustering (FSBC) for HT-SELEX data. FSBC was designed to estimate clusters by searching various lengths of over-represented strings as target binding regions. FSBC was also designed for fast calculation with search space reduction from a single round, typically the final round, of HT-SELEX data considering imbalanced nucleobases of the aptamer selection process. The calculation time and clustering accuracy of FSBC were compared with those of four conventional clustering methods, FASTAptamer, AptaCluster, APTANI, and AptaTRACE, using HT-SELEX data (>15 million oligonucleotide sequences). FSBC, AptaCluster, and AptaTRACE could complete the clustering for all sequence data, and FSBC and AptaTRACE performed higher clustering accuracy. FSBC showed the highest clustering accuracy and had the second fastest calculation speed among all methods compared.

**Conclusion:**

FSBC is applicable to a large HT-SELEX dataset, which can facilitate the accurate identification of groups including aptamer candidates.

**Availability of data and materials:**

FSBC is available at http://www.aoki.ecei.tohoku.ac.jp/fsbc/.

## Background

Systematic evolution of ligands by exponential enrichment (SELEX) is an experimental method for identifying aptamers, which bind to specific target molecules with high affinity and specificity [1, 2]. SELEX is an iterative method with multiple rounds for the enrichment of aptamers from the initial oligonucleotide random library. Each round consists of selection with target molecules and amplification with polymerase chain reaction(PCR). Aptamers are RNA or short single-stranded DNA molecules, which fold into a three-dimensional structure and bind different types of target molecules such as proteins [3], small molecules [4], toxins [5], ions [6], and cell surfaces [7]. Owing to the wide variety of possible target molecules, aptamers are commonly used for therapeutics [8], clinical diagnostics [9], the high-throughput multi-protein measurement [10], imaging [11], and biosensors [12].

**Fig. 1:**
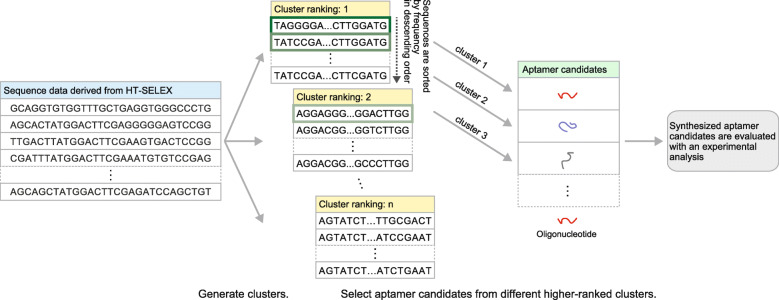
Procedure from obtaining clustering results to experimental analysis. HT-SELEX sequence data are grouped into different clusters according to cluster ranking. Sequences with a high frequency from high-ranked clusters are synthesized and evaluated for binding affinity with experimental analysis

A next-generation sequencing (NGS), which was originally developed for whole-genome sequencing, is available for analysis of large oligonucleotide pools obtained by SELEX to acquire an enormous sequence dataset for predicting aptamer candidates. This combined use of SELEX and NGS is referred to as high-throughput SELEX (HT-SELEX). It is not reasonable to evaluate the binding affinity with all observed sequences from NGS. In general, dozens of candidate aptamers are selected from the HT-SELEX data for evaluation with experimental analysis considering cost and time-consuming. In other words, the list of dozens of candidate aptamers is required from HT-SELEX data for evaluation with experimental analysis. Clustering for HT-SELEX data is an effective process to identify the sequence groups which are related to aptamer candidates, or noise sequences such as non-specific binding sequences, bead-binders, and PCR biased sequences which are easy to be enriched by PCR. Clustering is also useful to identify different types of aptamers such as different binding epitopes and for understanding the diversity and enrichment of oligonucleotide sequence pools. Figure 1 describes the typical procedure of selecting different types of aptamer candidates from the clustering results for binding verification with experimental analysis.

Several clustering methods have been developed for HT-SELEX data to date, including FASTAptamer [13], AptaCluster [14, 15], APTANI [16], and AptaTRACE [17]. FASTAptamer generates clusters based on Levenshtein-distance (LD) which represents the full length of sequence similarity with highly ranked sequences. AptaCluster first roughly groups sequences with local sensitive hashing (LSH) and then generates clusters with the short *k*-mer sequence similarity. APTANI and AptaTRACE identify clusters with short motifs considering the nucleic acid secondary structure. APTANI estimates motifs from a single round of SELEX data whereas AptaTRACE estimates motifs by tracing the changes of frequency between multiple rounds.

It is often observed that the most enriched sequence does not show the binding affinity to the target molecules. These noise sequences are likely to be generated by PCR bias (some oligonucleotide molecules are easy to be enriched by PCR) or non-specific binding of other molecules such as beads with charge effect. Typically, aptamers harbor a specific sequence region, which is necessary for binding to the target molecules, although noise oligonucleotide sequences generally do not include such a target binding region. Hence, determining the sequence clusters with such a target binding region could be an effective approach to choose aptamer candidates. The length of the target binding region varies according to the target molecules, epitopes, and/or binding styles. Thus, estimating target binding regions with different lengths is required. Although AptaTRACE was designed for detecting the candidate motifs as target binding regions, it has a limitation of the length of motifs and requires multiple rounds of SELEX data, which increases the sequencing cost.

To overcome these limitations, we developed the fast string-based clustering (FSBC) method. FSBC estimates clusters considering different lengths of over-represented strings as target binding regions. FSBC was also designed for fast calculation with search space reduction of over-represented strings using only a single round of HT-SELEX data, especially in the final round of SELEX, considering the imbalance of nucleobases of the aptamer selection process. FSBC implemented with R [18] is available at http://www.aoki.ecei.tohoku.ac.jp/fscb/.

## Methods

### Overview of the clustering algorithm

**Fig. 2:**
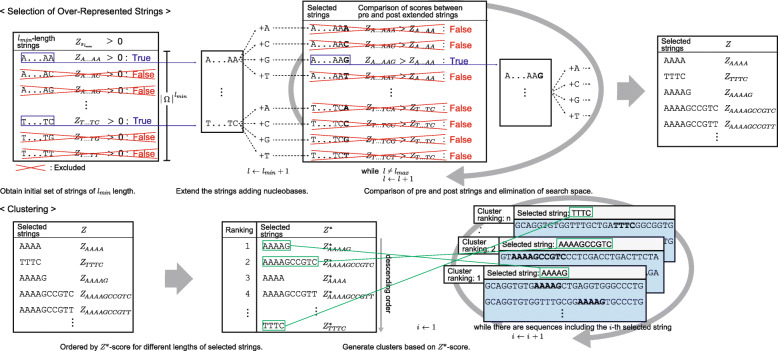
Outline of fast string-based clustering (FSBC). The algorithm includes over-represented string selection and clustering based on selected strings. The upper panel shows the selection of over-represented strings after minimizing the search space and comparing string scores (*Z*-scores) of pre- and post- extended strings. The lower panel shows the clustering based on selected strings ranked according to the *Z*^∗^-score, which is normalized *Z*-score for strings of different lengths

FSBC is composed of two parts: selection of over-represented strings with different lengths and sequence clustering based on the selected over-represented strings. For over-represented string selection, we propose a new score calculation method that considers the imbalanced ratios of nucleobases due to the selection process of SELEX. Figure 2 shows the outline of the FSBC algorithm.

### String score definition

For a set of nucleobases *Ω*={A,C,G,T(U)}, which represents adenine, cytosine, guanine, and thymine/uracil, respectively, the probability of each nucleobase is given as *p*_*j*_,(*j*∈*Ω*), the string is *s*, the length of the string is |*s*|, and the number of nucleobases in the string is *n*_*s*,*j*_. The probability of an *L*-mer oligonucleotide including string *s*, *P*_*s*,*L*_, is then described as the following recurrent equation:

1 $$ \begin{aligned} P_{s, L} = P_{s, L - 1} + Q \left (1 - P_{s, L - |s|} - \sum\limits_{t \in \mathcal T} q^{-1} \left(P_{s, L - |s| + |t|} - P_{s, L - |s| + |t| - 1}\right) \right), \\ \end{aligned}  $$

$$ Q = \prod\limits_{j \in \Omega} p_{j}^{n_{s, j}}, \quad q = \prod\limits_{j \in \Omega} p_{j}^{n_{t, j}}, \quad L \ge l, \notag  $$

where $\mathcal T$ is a set of self-overlapping regions of *s*, and $n_{t, j}, (t \in \mathcal T)$ is the number of nucleobases of the self-overlapping regions. For example, if string *s* is “ATATA”, the set of self-overlapping regions $\mathcal T_{\text {ATATA}}$ is {A, ATA}. If *L*<|*s*|, then *P*_*s*,*L*_=0. The terms *P*_*s*,*L*−1_, *Q*, *QP*_*s*,*L*−|*s*|_, and $Q \sum _{t \in \mathcal T} q^{-1} \left (P_{s, L - |s| + |t|} - P_{s, L - |s| + |t| - 1}\right)$ represent the probability that a sequence has the string from 1 to *L*−|*s*|−1, a sequence has the string at *L*−|*s*|, a sequence has the string both from 1 to *L*−|*s*|−1 and at *L*−|*s*|, and a sequence has the string at the self-overlapping position, respectively. Figure S1 shows a graphical representation of Eq. (1). In stringology, the probability calculation is the same approach with “missing words in random text” [19], and the self-overlapping region is the same meaning with “string overlaps” [20].

The lengths of observed sequences obtained using NGS vary owing to insertions and/or deletions during the SELEX process. Stoltenburg and Strehiltz described that around 78% of sequences had an expected length of random regions, while the other 22% of sequences are different from the original length of random region [21]. Therefore, the probability *P*_*s*,*L*_ was adjusted for different lengths of sequences using the following equation:

2 $$ P_{s} = \frac{1}{N} \sum\limits_{i=1}^{N} P_{s, L_{i}},  $$

where *N* is the number of observed sequences and *L*_*i*_ is the length of the *i*-th sequence.

Let the frequency of observed sequences including string *s* be *F*_*s*_. *P*_*s*_ follows a binomial distribution. If *N* is a large enough number for *F*_*s*_, a random variable representing the difference between *F*_*s*_ and *P*_*s*_ normalized by the standard deviation of the binomial distribution then shows an approximate normal Gaussian distribution. Hence, the *Z*-score for string *s* is derived according to the following equation:

3 $$ Z_{s} = \frac{\frac{F_{s}}{N} - P_{s}}{\sqrt{\frac{P_{s} (1 - P_{s}) }{N}}}.  $$

### Selection of over-represented strings

Before selection of the over-represented strings, the probability of each nucleobase, $\hat p_{j}$, is estimated with the following equation:

4 $$ \hat p_{j} = \frac{n_{j}}{\sum_{i = 1}^{N} L_{i}}, \quad j \in \Omega,  $$

where *n*_*j*_ is the number of observed nucleobases. These estimated probabilities are then used for calculation of the *Z*-scores. Since the ratios of nucleobases in the SELEX pool can change owing to the systematic selection bias of SELEX, the *Z*-score is calculated based on the balance of nucleobases using Eqs. (1) – (4).

Over-represented strings with lengths ranging from *l*_*min*_ to *l*_*max*_ are selected while reducing the search space from all possible combinations by comparing *Z*-scores. Selection of over-represented strings is then conducted according to the following process:

1. Enumerate all *l*_*min*_-length strings and calculate their *Z*-scores. Exclude string whose *Z*-scores are less than 0.
2. Substitute *l*←*l*_*min*_.
3. Enumerate extended strings by adding a nucleobase and calculate their *Z*-scores. Exclude extended strings whose *Z*-scores are less than those of the pre-extended strings.
4. If *l*+1>*l*_*max*_, then finish the selection of over-represented strings.
5. Substitute *l*←*l*+1, and go to 3.

The algorithm for estimating over-represented strings reduces the search space by comparing of *Z*-scores between the post-extended and pre-extended strings. Thus, the number of selected strings, *m*, is much smaller than the exhaustive enumeration of all strings: $m \ll \sum _{l = l_{min}}^{l_{max}} |\Omega |^{l}$. This search space minimization provides a huge reduction in the calculation time for an HT-SELEX dataset.

### Clustering with selected over-represented strings

While extending the string length, the strings with higher Z-scores are selected for search space reduction. For evaluating the different lengths of strings equally, the normalization of the Z-score was performed. The normalized *Z*-score for string *s*, referred to as $Z_{s}^{*}$, is calculated with the following equation:

5 $$ Z_{s}^{*} = \frac{Z_{s} - \hat \mu_{|s|}}{\hat \sigma_{|s|}},  $$

where $\hat \mu _{|s|}$ and $\hat \sigma _{|s|}$ are the mean and standard deviation, respectively, of the *Z*-score of selected strings with length |*s*|. The strings are then ordered by *Z*^∗^. Parameters $\hat \mu _{|s|}$ and $\hat \sigma _{|s|}$ are estimated with only selected strings. Therefore, there are no guarantees of Gaussian distribution of *Z*^∗^. The clustering is then achieved according to the following process:

1. Substitute *i*←1.
2. Extract sequences including the *i*-th strings from the sequence dataset, where a set of extracted sequences is referred to as the *i*-th cluster. Remove extracted sequences from the sequence dataset.
3. If there are no sequences remaining, finish the clustering.
4. Substitute *i*←*i*+1, and go to step 2.

### Data

The publicly available whole-cell SELEX dataset of human embryonic stem cells [22] was used for comparing the calculation speed and clustering accuracy. The SELEX was finished at the fifth round and nineteen sequences were evaluated for binding affinity with flow cytometry. According to the binding evaluation, eight of nineteen sequences showed higher fluorescent intensity and those sequences were defined as target-binding sequences.

### Calculation time

The sequence data were filtered with different frequency cut-offs (1, 10, and 100) to vary the size of the dataset. The numbers of sequences included with frequency cut-offs of 1, 10, and 100 were 15,327,604 (4,381,160), 8,799,219 (156,587), and 4,947,522 (6,193) with 1, 10, and 100, respectively; the numbers of non-redundant sequences are indicated in parentheses.

The five different algorithms, namely FASTAptamer, AptaCluster, APTANI, AptaTRACE, and FSBC, were compared with respect to calculation time. The fifth round HT-SELEX data, which was the last round of SELEX, were used for FASTAptamer, AptaCluster, APTANI, and FSBC. The fourth and fifth round HT-SELEX data were used for AptaTRACE because AptaTRACE requires multiple rounds of HT-SELEX data.

FASTAptamer was performed with an edit distance option of 7 (according to the user guide), and the maximum cluster number was set to 100 to reduce the calculation time. AptaCluster was performed with the default options. The options for APTANI were no-filtering of frequency, fixed length for HT-SELEX data, and primer information for estimation of the secondary structure. There are no further options for reducing the calculation time except for frequency filtering; thus, we did not change any options for APTANI. AptaTRACE was performed with the background sequence option as 1,000 because AptaTRACE demonstrated the best accuracy with that parameter. The options of FSBC were *l*_*min*_=5 and *l*_*max*_=10.

FSBC was written in R [18] version 3.6.2 with Bioconductor packages [23], and other programs are provided with scripts and executable files. The computer specifications were as follows: OS Ubuntu 16.04 (Xenial Xerus) 64bit, Intel(R) Xeon(R) CPU E5-1650v4@3.60GHz, and 64 GB memory.

### Clustering accuracy

Filtered data (frequency ≥10) of the fifth round, which was the final round of the SELEX, was applied for comparing the accuracy of the clustering methods because FASTAptamer and APTANI did not complete the clustering with the entire sequence dataset. The same parameters indicated in the previous subsection for AptaCluster and APTANI were applied for evaluating the clustering accuracy. The option of the maximum number of clusters for FASTAptamer was not used for the evaluation of clustering accuracy. Changing the parameters of LD and motif length did not improve the accuracy of FASTAptamer and AptaCluster, respectively. For AptaTRACE, the background sequence option was set as 1,000 because AptaTRACE showed the highest accuracy with that option. The options for FSBC were *l*_*min*_={3,4,5} and *l*_*max*_=10. FSBC was also applied to the entire sequence dataset and the filtered data (frequency ≥100) to evaluate the potential bias of frequency filtering, and missing aptamer sequences due to the sequence frequency filtering.

The sequences with binding/non-binding information were sorted with cluster ranking for each method. For evaluating the cluster ranking and binding sequences, the receiver operating characteristic (ROC) curves were generated according to the order of cluster ranking with the binding information. The area under the curve (AUC) values were calculated based on the area of the ROC curves. FSBC was also applied to all of the sequence data from the third and fourth rounds of SELEX to evaluate the possibility of the detection of aptamers in early rounds.

### Comparison with exhaustive enumeration of strings

Due to the search space reduction, there are no guarantees that the top-ranked strings of exhaustive enumeration are included in the selected strings. Hence, we verified whether the top-ranked strings of exhaustive enumeration was included in the selected strings or not. The missing rate of the top ten ranked strings of exhaustive enumeration was also evaluated for each length.

## Results

### Calculation time

Table 1 shows the calculation time for each method and the dataset size. The first column shows the clustering methods, and the second to seventh columns represent the actual and CPU time for each size of dataset. Note that the calculation time of FASTAptamer includes the pre-processing time, which involves counting the frequency of sequences, before clustering.

**Table 1:** Clustering calculation time for each method with datasets of different sizes. Sequences (≥ 10) and sequences (≥ 100) represent filtered data with frequency cutoff. DNF indicates did not finish. DNF ^1^: FASTAptamer did not complete the calculation for the entire sequence dataset in 7 days. DNF ^2^: APTANI showed a calculation error after the prediction of the secondary structure, which took 25 h

	All sequences		Sequences (≥ 10)		Sequences (≥ 100)	
Method	Real time	CPU time	Real time	CPU time	Real time	CPU time
FASTAptamer	DNF ^1^	DNF ^1^	5 h 16 m 4 s	5 h 16 m 3 s	10 m 40 s	10 m 40 s
AptaCluster	3 m 45 s	4 m 9 s	33 s	26 s	28 s	17 s
APTANI	DNF ^2^	DNF ^2^	32 m 52	34 m 59 s	1 m 47 s	1 m 20 s
AptaTRACE	71 h 38 m 35 s	246 h 15 m 12 s	1 h 1 m 17 s	2 h 2 m 50 s	3 m 52 s	5 m 44 s
FSBC	4 h 40 m 51 s	4 h 40 m34 s	9 m 25 s	9 m 17 s	51 s	46 s

AptaCluster showed the fastest calculation time for clustering, followed by FSBC. However, FASTAptamer was the slowest of the five methods and did not complete the clustering of the entire dataset in 7 days, even when changing the clustering number option to “-c 100” to reduce the calculation time. APTANI also could not complete the calculation for the entire dataset due to an error after the secondary structure prediction, which required 25 h. AptaTRACE calculated clustering with parallel computing, hence the real-time was much smaller than CPU time.

### Accuracy

The clustering result for each algorithm is shown in Table 2. The columns indicate the oligonucleotide sequences excluding both ends of the primers, sequence ID, ranking of frequency, frequency, binding information, and cluster ranking for each method. AptaCluster has a two-ranking system for clustering, including frequency and diversity, corresponding to the frequency of sequences in the cluster and the number of non-redundant sequences in the cluster, respectively. APTANI does not include any functions for ordering clusters; thus, we used frequency and diversity for this purpose as performed by AptaCluster. The binding information was already defined by the verification of experimental analysis using flow cytometry [22]. Sequence IDs, seq1 to seq8 are defined as binding sequences whereas sequence IDs seq9 to seq19 are not the binding sequences. Sequence ID seq8 was not included since it was filtered out based on the frequency cut-off before clustering. The strings selected by FSBC are underlined and in uppercase in the table. The order of sequences in Table 2 is based on the ranking of the frequency on binding/non-binding sequences. FASTAptamer, AptaCluster (frequency/diversity), APTANI (frequency/diversity), AptaTRACE and FSBC estimated 2,380, 136,350, 2,348, 13, and 155 clusters, respectively.

**Table 2:** Cluster ranking. AptaCluster (Freq.), AptaCluster (Div.), and APTANI (Freq.), APTANI (Div.) represent the cluster ranking of frequency and diversity (the number of non-redundant sequences) of AptaCluster and APTANI, respectively. Sequences with a frequency of less than 10 were excluded before the clustering analysis. Because FASTAptamer and APTANI did not finish with all sequence data. *: This sequence is filtered as the frequency is less than 10. **: The ranking of clusters is tied; however, the sequences are not grouped in the same cluster. ***: These sequences did not include any motifs estimated by AptaTRACE, thus the sequences are not grouped into any clusters

Sequence information					Cluster ranking						
Sequence	ID	Ranking	Frequency	Binding	FASTAptamer	AptaCluster	AptaCluster	APTANI	APTANI	AptaTRACE	FSBC
						(Freq.)	(Div.)	(Freq.)	(Div.)		(*l*_*min*_=5)
aggaggggGACTTaggactgggtttaggg	seq1	6	92237	Yes	6	7	5	7	870	1	5
agggTATGGACTTCgacgtctcggctgaa	seq2	24	20057	Yes	15	17	15	15	699	1	1
cgcacaggaaggTATGGACTTCgacgttt	seq3	63	8750	Yes	24	64	65	58	290	1	1
ggTATGGACTTCgacgtcttctgacctaa	seq4	82	6753	Yes	15	81	72	68	2188	1	1
gaaaTATGGACTTCgatacgccggctgag	seq5	255	1483	Yes	60	229	112740	102	626 ^∗∗^	1	1
agtatctatccGACTTggatttacgttcg	seq6	8459	84	Yes	546	9921	28056	1993	626 ^∗∗^	NA***	5
tatccGACTTggatggctgagcaaggcta	seq7	100914	15	Yes	731	94490	125262	2038	626 ^∗∗^	5	5
aggaggggGACTTaggactgggtttatga^∗^	seq8	281478	4	Yes	NA	NA	NA	NA	NA	NA	NA
gcaggtgtggtttgctgaggTGGGCCctg	seq9	1	583447	No	1	1	2	1	125	4	26
tttggtttgctgTATGGtgggctctgtta	seq10	8	70095	No	7	8	10	8	916 ^∗∗^	4	16
gtgagggtgAGGACaggttagcgtggtgg	seq11	10	51669	No	9	11	9	16	916 ^∗∗^	7	54
ggtgaggcgGACGTatcttttagcaaatc	seq12	12	45038	No	10	12	13	13	520	11	41
tcgcttgaacggggaactactccaGACGT	seq13	23	20380	No	14	21	23	45	2270	NA***	41
gTGGGCgcacttagacggggtgatcgtaa	seq14	375	831	No	75	335	76783	387	1739	NA***	37
ACTTAtttgtcttaagtggcgggtcaatg	seq15	398	771	No	78	238	556	460	2188	8	47
gggtccCTTCGgggtgacgatggtatcta	seq16	520	504	No	107	466	120874	1758	2253	NA***	11
ggtGTGGGgagggtcgtattgtgtcctgt	seq17	3847	126	No	388	4568	59849	92	1	5	66
cttatttgtgtttagtggcgggcGTTTGt	seq18	29324	41	No	50	539	110	44	323	NA***	92
ctatttgTTCTAgtggcggtcatctaagg	seq19	44000	31	No	50	9134	4859	2043	2253	NA***	88

Among the five methods, only FSBC and AptaTRACE provided a top-ranked cluster that included binding sequences. By contrast, the top-ranked clusters obtained with FASTAptamer, AptaCluster (frequency), and APTANI, and the second top-ranked cluster obtained by AptaCluster (diversity) included the top-ranked sequence of “frequency”, which did not show binding ability. Similarly, APTANI (diversity) yielded a top-ranked cluster including sequence ID seq17, which also did not bind to the target molecules. FASTAptamer, AptaCluster (frequency), AptaCluster (diversity), APTANI (frequency), and APTANI (diversity) showed 6, 7, 5, 7, and 290 as the highest ranked clusters including binding sequences, respectively, and these ranks were all lower than those with non-binding sequences. FSBC and AptaTRACE grouped all binding sequences from sequence ID seq1 to seq7 into two clusters with cluster ranks 1 and 5. However, AptaTRACE missed sequence ID seq6, and sequence ID seq7 was grouped with sequence ID seq17 which did not show the binding affinity. FASTAptamer grouped sequence ID seq2 and seq4 into the same cluster, which was ranked fifteenth. APTANI (diversity) showed the same cluster ranking from sequence ID, seq5 to seq8; however, these ranks were simply tied but the sequences did not group in the same cluster. AptaCluster (frequency/diversity) and APTANI (frequency) did not group any binding sequences into the same cluster. Table S1 shows the same result of FSBC with all sequences (no-filtering with frequency cutoff) and filtered data (≥100) under the option *l*_*min*_=5. Similar to the result in Table 2, all binding sequences were in the higher-ranked clusters rather than in the clusters ranked with non-binding sequences.

FSBC selected a total of 1,003 strings, and the top 24 strings are shown in Table S2. The selected over-represented strings “ATGGACTTCGG” and “GACTT”, ranked 1 and 12, respectively, were included in cluster 1 and 5 in Table 2. The selected string “GACTT” is a part of string “ATGGACTTCGG”. The distribution of the *Z*-scores and *Z*^∗^-scores of the selected strings for each length of string is shown in Figure S2.

**Fig. 3:**
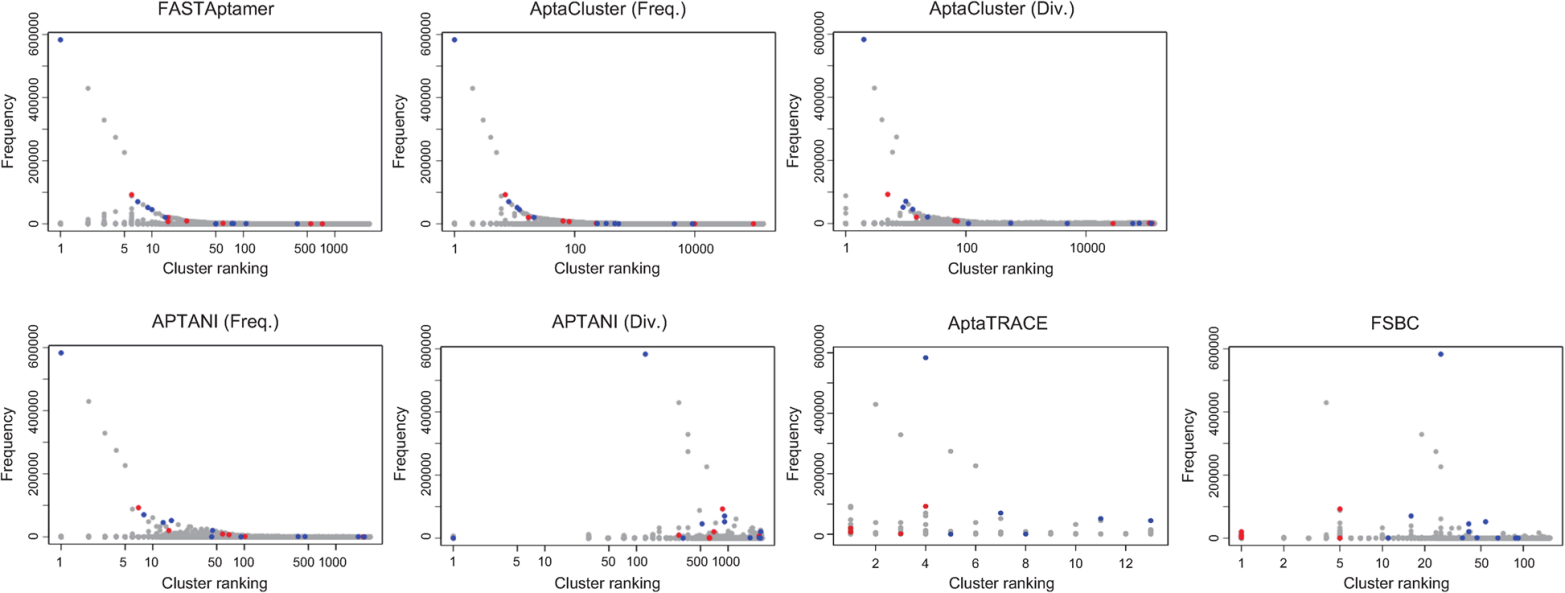
Relation between cluster ranking and frequency of sequences. Binding, non-binding, and non-evaluated sequences are shown as red, blue, and gray dots, respectively

The relation between cluster ranking and frequency of oligonucleotide sequences with each method is displayed in Fig. 3, in which the red, blue, and gray dots represent binding, non-binding, and non-evaluated sequences to the target molecules, respectively. The top-ranked clusters obtained by FASTAptamer, AptaCluster (frequency), and APTANI (frequency) included the non-binding sequence of the highest frequency. AptaCluster (diversity) and APTANI (diversity) included the non-binding sequence of the highest frequency in higher ranked cluster than those including binding sequences. By contrast, FSBC and AptaTRACE grouped the binding sequences with lower frequencies in the top-ranked cluster.

**Fig. 4:**
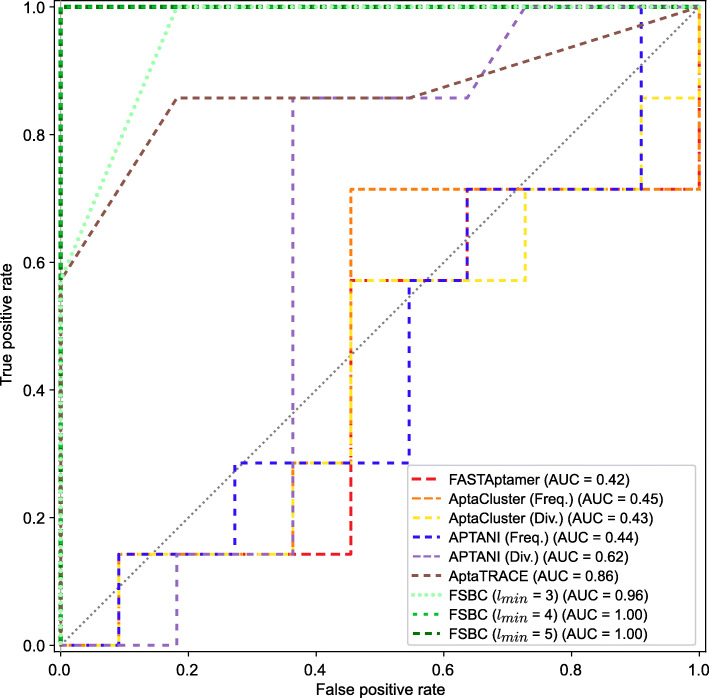
Receiver operating characteristic (ROC) curves of different clustering methods. “Freq.” and “Div.” in the parentheses (after AptaCluster and APTANI) indicate the cluster ranking with frequency and diversity (the number of non-redundant sequences) in the cluster for the respective method. AUC indicates the area under the curve

The ROC curve and AUC value for each clustering method are displayed in Fig. 4. FSBC with options *l*_*min*_=4 and *l*_*min*_=5 clearly distinguished binding from non-binding sequences, i.e. the AUC value equals to 1. The AUC value was slightly lower (0.96) when the FSBC options *l*_*min*_=3 were applied, because some non-binding sequences were grouped into the same binding cluster. AptaTRACE also demonstrates a higher AUC value because AptaTRACE detected the target binding regions in the higher-ranked clusters. However, the other clustering methods resulted in lower AUCs because non-binding sequences with high frequency were included in the higher-ranked clusters. FSBC with option *l*_*min*_=5 also showed that the AUC value equals to 1 for all sequence data and filtered data (frequency ≥ 100) in Table S1. The clustering results for third- and fourth-round data are summarized in Table S3 and Table S4, respectively. FSBC could identify aptamer sequences in the third and first clusters from third-and fourth-round data. AUC values for the third and fourth rounds are 0.89 and 1, respectively.

### Comparison with exhaustive enumeration of strings

The top-ranked over-represented string of exhaustive enumeration was included in the selected strings for each length. The missing rate of the top 10 ranked strings of exhaustive enumeration for each length is shown in Table S5. Top 10 ranked 10-mer strings of the exhaustive enumeration include 6 selected strings. Thus, the missing rate of the 10-mer string is 0.4.

## Discussion

Our newly developed algorithm for clustering, FSBC, showed the second fastest calculation speed with HT-SELEX data. AptaCluster displayed a remarkably fastest calculation time. FASTAptamer and APTANI could not complete the clustering for all of the sequence data; hence, only FSBC, AptaCluster, and AptaTRACE are available for applications with a real HT-SELEX dataset. FSBC selected a total of 1,003 strings, which was much smaller than all exhaustive enumeration of strings: $\sum _{i = 5}^{10} 4^{i} = 1,397,760$. The ratio of the number of selected strings over all combinations is 1,003/1,397,760=0.0007175767. Hence, the minimization of the search space was an effective method for finding over-represented strings of longer lengths such as 10-mer. FSBC was designed for handling a single-round sequence data from SELEX. This approach could also be helpful to reduce the sequencing cost and the calculation time compared to other methods such as MPBind [22] and AptaTRACE[17], which require multiple rounds of sequence data from SELEX pools.

Importantly, FSBC and AptaTRACE distinguished binding sequences as high-ranked clusters, whereas the other clustering methods categorized non-binding sequences with high frequency under high-ranked clusters. This demonstrates that FASTAptamer, AptaCluster, and APTANI are more sensitive to the frequency of sequences rather than to enrichment of over-represented strings. Thus, if the SELEX pool contains numerous non-binding sequences due to PCR bias, FASTAptamer, AptaCluster, and APTANI might place these PCR biased sequences in the high-ranked clusters. The sequencing data used for the current study includes enriched strings among the binding sequences, and FSBC and AptaTRACE could accurately detect these strings as the estimated target binding region. AptaTRACE detected binding sequences with higher-ranked clusters, however, the cluster of rank 5 includes both binding and non-binding sequences. Consequently, FSBC showed a better result for cluster ranking in this study.

This proposed string score calculation method can be extended to combine with other outcomes. In this study, we defined the outcome according to nucleobases: *Ω*_*nucleobase*_={A,C,G,*T*(*U*)}. However, other outcomes can also be defined, such as the oligonucleotide secondary structure: *Ω*_*structure*_={H,B,S,M,E,I,G}, which represent the structure of the hairpin loop, bulge, stem, multi-loop, external loop, internal loop, and G-quadruplex, respectively. A set of outcomes can be extended as *Ω*=*Ω*_*nucleobase*_×*Ω*_*structure*_. If the set is extended to include the secondary structure, FSBC is available for searching over-represented strings with a specific secondary structure. However, the calculation time will also increase with increasing the number of elements of *Ω*. Hence, to obtain the fastest calculation with FSBC, *Ω*=*Ω*_*nucleobase*_ is the reasonable outcome. This string scoring method can also be used for other types of sequence analysis such as for amino acid sequences. In other words, if *Ω* is defined based on amino acids, Eq. (1) can be used for finding over-represented strings among amino acid sequences.

FSBC does not consider insertion/deletions or degenerated nucleobases, because the method was designed to reduce the calculation time to enable estimating longer over-represented strings in a huge dataset. Since the size of clusters is much smaller than the size of the entire sequence dataset, other motif-estimating methods such as MEME [24] can be used for more accurate estimation of candidate motifs.

Due to a lack of publicly accessible HT-SELEX data with binding information, only one HT-SELEX dataset was used. Sequence data could differ depending on the target molecules, SELEX methods, and initial bias of SELEX. Hence, the evaluation with other HT-SELEX data should be performed. After there will be enough dataset of HT-SELEX data publicly available for evaluation, the clustering methods need to be summarized. Moreover, only a single clustering method cannot cover all types of SELEX datasets. Thus, the most suitable clustering approach is to compare and summarize the results of different clustering methods.

## Conclusion

We proposed a new and rapid string-based clustering method for HT-SELEX data. Our clustering method could complete the calculation from a huge dataset in a reasonable time, even though the method is designed to estimate longer over-represented strings such as 10-mer. Importantly, our clustering method could identify enriched strings that were included in binding sequences estimated as the target binding region of the aptamer. Overall, FSBC could be a helpful method to effectively identify aptamers with HT-SELEX data.

## Supplementary information

**Additional file 1** The supplementary document includes supplementary tables (Tables S1 to S5) and figures (Figures S1 and S2).

## Data Availability

FSBC was implemented with R version 3.6.2 and is available at http://www.aoki.ecei.tohoku.ac.jp/fsbc/.

## Abbreviations

FSBC: Fast string-based clustering
SELEX: Systematic evolution of ligands by exponential enrichment
HT-SELEX: High-throughput systematic evolution of ligands by exponential enrichment
NGS: Next-generation sequencing
LD: Levenshtein-distance
LSH: Locality sensitive hashing
ROC: Receiver operating characteristic
AUC: Area under the curve
PCR: Polymerase chain reaction
