# Supplementary material for: FSBC: fast string-based clustering for HT-SELEX data
Source: BMC Bioinformatics. 2020 Jun 24;21:263. doi: 10.1186/s12859-020-03607-1 (PMC7313139; doi:10.1186/s12859-020-03607-1)
Supplement: Supplementary file 1 — Additional file 1 The supplementary document includes supplementary tables (Tables S1 to S5) and figures (Figures S1 and S2). [file 12859_2020_3607_MOESM1_ESM.pdf]

Supplementary Document of
FSBC: Fast string-based clustering for HT-SELEX data

Shintaro Kato[1,2], Takayoshi Ono[2], Hirotaka Minagawa[1], Katsunori Horii[1], Ikuo Shiratori[1], Iwao Waga[1], Koichi Ito[2], and Takafumi Aoki[2]

[1]NEC Solution Innovators, Ltd.
[2]Graduate School of Information Sciences, Tohoku University

Figure S1 shows the graphical explanation of the probability of sequence that includes a specific string.

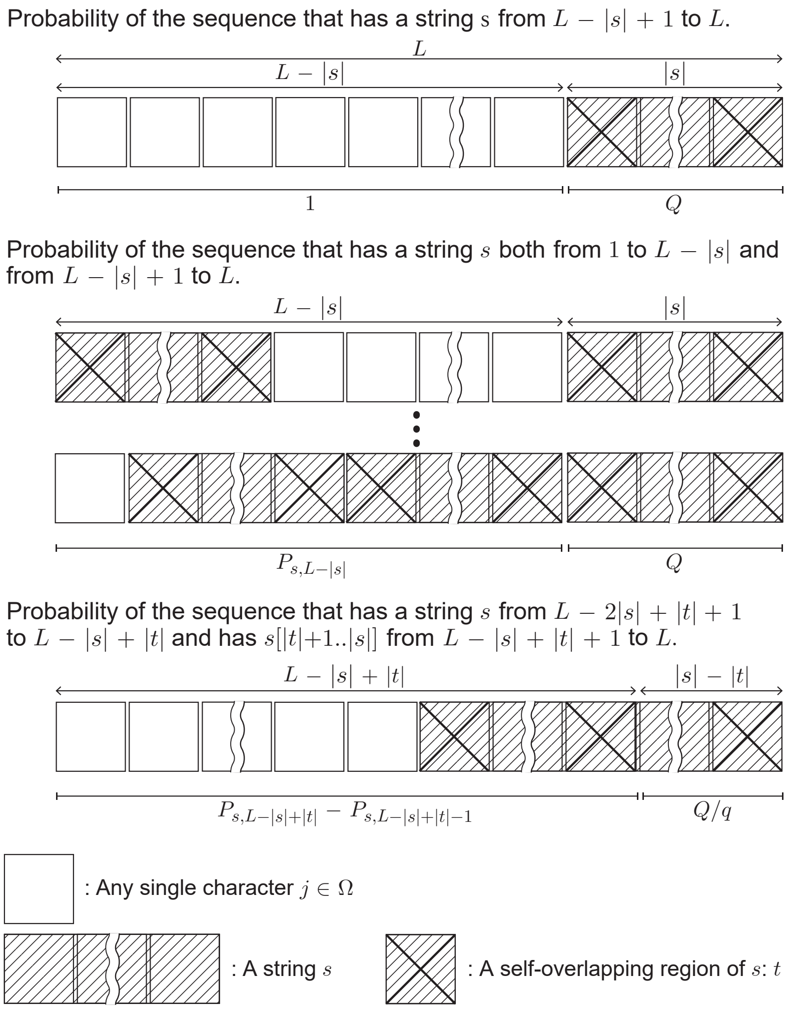

Figure S1: Graphical explanation of the probability of sequence including a specific string

Table S1 shows the clustering result of FSBC with all sequences and filtered data (frequency $\geq$ 100) with the fifth round data. Columns are sequence, the ranking of frequency, frequency, binding information, cluster ranking for all sequences, cluster ranking for filtered data ($\geq$ 100). With both datasets, FSBC shows binding sequences are grouped into the higher ranked clusters and non-binding sequences are grouped into the lower-ranked clusters.

Table S1: FSBC clustering result of all sequences and filtered seuqneces (frequency $\geq$ 100) with the fifth round data

Sequence	Ranking	Frequency	Binding	All sequences	Frequency ($\geq$ 100)
AGGAGGGGGACTTAGGACTGGGTTTAGGG	6	92237	Yes	9	2
AGGGTATGGACTTCGACGTCTCGGCTGAA	24	20057	Yes	1	1
CGCACAGGAAGGTATGGACTTCGACGTTT	63	8750	Yes	1	1
GGTATGGACTTCGACGTCTTCTGACCTAA	82	6753	Yes	1	1
GAAATATGGACTTCGATACGCCGGCTGAG	255	1483	Yes	1	1
AGTATCTATCCGACTTGGATTTACGTTCG	8459	84	Yes	9	NA
TATCCGACTTGGATGGCTGAGCAAGGCTA	100914	15	Yes	9	NA
AGGAGGGGGACTTAGGACTGGGTTTATGA	281478	4	Yes	9	NA
GCAGGTGTGGTTTGCTGAGGTGGGCCCTG	1	583447	No	25	15
TTTGGTTTGCTGTATGGTGGGCTCTGTTA	8	70095	No	17	8
GTGAGGGTGAGGACAGGTTAGCGTGGTGG	10	51669	No	59	28
GGTGAGGCGGACGTATCTTTTAGCAAATC	12	45038	No	45	25
TCGCTTGAACGGGGAACTACTCCAGACGT	23	20380	No	45	25
GTGGGCGCACTTAGACGGGGTGATCGTAA	375	831	No	40	24
ACTTATTTGTCTTAAGTGGCGGGTCAATG	398	771	No	68	24
GGGTCCCTTCGGGGTGACGATGGTATCTA	520	504	No	13	5
GGTGTGGGGAGGGTCGTATTGTGTCCTGT	3847	126	No	74	41
CTTATTTGTGTTTAGTGGCGGGCGTTTGT	29324	41	No	182	NA
CTATTTGTTCTAGTGGCGGTCATCTAAGG	44000	31	No	170	NA

Table S2 shows the selected over-represented strings with the fifth round data by FSBC. The columns are selected strings, frequency of selected strings, the ratio of selected strings, expected probability of selected strings, Z-score, Z^*-score, length of selected strings, and ranking of selected strings with Z^*-score. Selected strings of rank 1 and 12 were used for clusters of ranking 1 and 5, respectively. Selected strings of rank 1 and 12 were "ATGGACTTCG" and "GACTT", respectively. Thus, the selected string of rank 12 is a part of the selected string of rank 1. Hence, the sequences belonging to clusters of ranking 1 and 5 could show similar binding style.

Figure S1 describes the distribution of Z-score (left) and Z^*-score (right) for each length of the selected strings. Some selected strings from length 5 to length 10 show a higher score comparing to the other strings.

Table S2: Estimated candidate motifs with the fifth round data

Motif	Frequency	Ratio	Probability	Z	Z*	Length	Ranking
TATGGACTTC	5121621	0.58	2.04e-05	382174.26	6.65	10	1
ATGGACTTC	5208703	0.59	7.72e-05	199781.72	6.46	9	2
ATGGACTTCG	5200405	0.59	2.29e-05	366247.93	6.34	10	3
TGGACTTCGA	5136257	0.58	2.29e-05	361730.02	6.26	10	4
ACTTC	5716032	0.65	1.57e-02	15119.69	6.22	5	5
ACTTCGA	5146096	0.58	9.79e-04	55393.70	6.22	7	6
GACTTCGA	5145894	0.58	2.92e-04	101556.41	6.09	8	7
GGACTTCGA	5144262	0.58	8.67e-05	186221.12	5.98	9	8
GACTTC	5708715	0.65	4.72e-03	27883.46	5.92	6	9
ACTTCG	5244713	0.60	4.72e-03	25600.50	5.40	6	10
TGGACTTC	5216145	0.59	3.74e-04	90868.33	5.39	8	11
GACTT	6319740	0.72	2.50e-02	13159.73	5.35	5	12
CTTCGA	5147263	0.58	4.72e-03	25121.04	5.28	6	13
TGGACTTCG	5203766	0.59	1.11e-04	166301.38	5.28	9	14
TATGGACTT	5122495	0.58	1.10e-04	164636.67	5.22	9	15
GGACTTC	5249863	0.60	1.41e-03	47063.58	5.20	7	16
GACTTCG	5244085	0.60	1.41e-03	47015.09	5.19	7	17
ATGGACTT	5209664	0.59	4.15e-04	86139.64	5.08	8	18
GGACTTCG	5237243	0.60	4.20e-04	86107.20	5.07	8	19
TATGGACT	5122841	0.58	4.15e-04	84705.02	4.98	8	20
ATGGACT	5210219	0.59	1.56e-03	44326.53	4.87	7	21
TATGGAC	5123903	0.58	1.56e-03	43590.24	4.78	7	22
ATGGAC	5211685	0.59	5.88e-03	22760.87	4.74	6	23
GGACTT	5805259	0.66	7.53e-03	22373.14	4.65	6	24

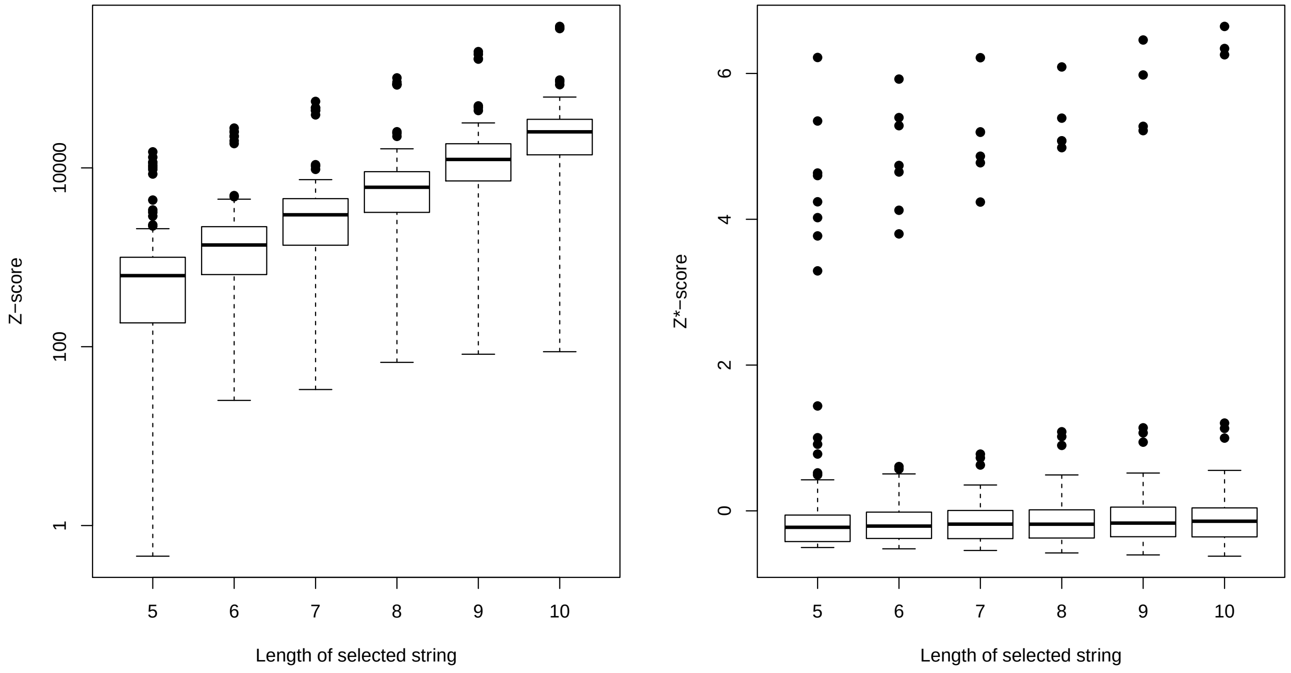

Figure S2: Distribution of Z-score and Z^*-score with different lengths of selected strings

Table S3 and S4 displays the clustering result by FSBC for the third and fourth round of SELEX data. The AUCs for the third round and fourth round are 0.89 and 1, respectively.

Table S3: FSBC clustering result of the third round

Sequence	Ranking	Frequency	Ratio	Binding	Cluster ranking
AGGAGGGGGACTTAGGACTGGGTTTAGGG	38	10	4.02e-07	Yes	3
CGCACAGGAAGGTATGGACTTCGACGTTT	82	5	2.01e-07	Yes	5
AGGGTATGGACTTCGACGTCTCGGCTGAA	457	3	1.21e-07	Yes	5
GAAATATGGACTTCGATACGCCGGCTGAG	10134	2	8.05e-08	Yes	5
AGTATCTATCCGACTTGGATTTACGTTCG	474449	1	4.02e-08	Yes	34
GGTATGGACTTCGACGTCTTCTGACCTAA	474449	1	4.02e-08	Yes	5
TATCCGACTTGGATGGCTGAGCAAGGCTA	NA	NA	NA	Yes	NA
AGGAGGGGGACTTAGGACTGGGTTTATGA	NA	NA	NA	Yes	NA
GCAGGTGTGGTTTGCTGAGGTGGGCCCTG	2	89	3.58e-06	No	25
TTTGGTTTGCTGTATGGTGGGCTCTGTTA	2	89	3.58e-06	No	25
GGTGAGGCGGACGTATCTTTTAGCAAATC	47	8	3.22e-07	No	11
TCGCTTGAACGGGGAACTACTCCAGACGT	59	6	2.41e-07	No	45
GTGAGGGTGAGGACAGGTTAGCGTGGTGG	10134	2	8.05e-08	No	16
ACTTATTTGTCTTAAGTGGCGGGTCAATG	474449	1	4.02e-08	No	36
GTGGGCGCACTTAGACGGGGTGATCGTAA	NA	NA	NA	No	NA
GGGTCCCTTCGGGGTGACGATGGTATCTA	NA	NA	NA	No	NA
GGTGTGGGGAGGGTCGTATTGTGTCCTGT	NA	NA	NA	No	NA
CTTATTTGTGTTTAGTGGCGGGCGTTTGT	NA	NA	NA	No	NA
CTATTTGTTCTAGTGGCGGTCATCTAAGG	NA	NA	NA	No	NA

Table S4: FSBC clustering result of the fourth round

Sequence	Ranking	Frequency	Ratio	Binding	Cluster ranking
AGGAGGGGGACTTAGGACTGGGTTTAGGG	15	6837	2.71e-04	Yes	2
AGGGTATGGACTTCGACGTCTCGGCTGAA	53	1639	6.49e-05	Yes	1
CGCACAGGAAGGTATGGACTTCGACGTTT	112	926	3.67e-05	Yes	1
GGTATGGACTTCGACGTCTTCTGACCTAA	130	747	2.96e-05	Yes	1
GAAATATGGACTTCGATACGCCGGCTGAG	308	306	1.21e-05	Yes	1
AGTATCTATCCGACTTGGATTTACGTTCG	14892	39	1.54e-06	Yes	8
TATCCGACTTGGATGGCTGAGCAAGGCTA	109981	13	5.15e-07	Yes	8
AGGAGGGGGACTTAGGACTGGGTTTATGA	NA	NA	NA	Yes	NA
GCAGGTGTGGTTTGCTGAGGTGGGCCCTG	1	66644	2.64e-03	No	32
TTTGGTTTGCTGTATGGTGGGCTCTGTTA	2	59338	2.35e-03	No	12
GGTGAGGCGGACGTATCTTTTAGCAAATC	27	4259	1.69e-04	No	18
TCGCTTGAACGGGGAACTACTCCAGACGT	36	3103	1.23e-04	No	66
GTGAGGGTGAGGACAGGTTAGCGTGGTGG	90	1121	4.44e-05	No	39
ACTTATTTGTCTTAAGTGGCGGGTCAATG	239	405	1.60e-05	No	40
GTGGGCGCACTTAGACGGGGTGATCGTAA	1463	89	3.52e-06	No	55
CTATTTGTTCTAGTGGCGGTCATCTAAGG	10173	45	1.78e-06	No	40
GGGTCCCTTCGGGGTGACGATGGTATCTA	23905	32	1.27e-06	No	10
CTTATTTGTGTTTAGTGGCGGGCGTTTGT	54930	21	8.32e-07	No	40
GGTGTGGGGAGGGTCGTATTGTGTCCTGT	120767	12	4.75e-07	No	39

Table S5 shows the comparison between exhaustive enumeration and selected strings. The first column shows the length of the string, the second column represents the top-ranked string of exhaustive enumeration is included in the selected strings. The third column represents the number of selected strings which is in the top 10 ranked strings of exhaustive enumeration.

Table S5: Comparison between exahstive enumeration and selected strings

Length	Includes Top Ranked String	Frequency
6	Yes	7
7	Yes	7
8	Yes	7
9	Yes	6
10	Yes	6
